# ASAH2 deficiency affects sphingolipid homeostasis and neuromotor control, causing a progressive neurological disorder

**DOI:** 10.1016/j.xhgg.2026.100587

**Published:** 2026-03-10

**Authors:** Marcello Scala, Ranjan K. Sahu, Mariasavina Severino, Monica Traverso, Michele Iacomino, Marina Pedemonte, Filippo Santorelli, Stefano Tozza, Federico Zara, Chiara Fiorillo, Hyung-lok Chung

**Affiliations:** 1 Department of Neurosciences, Rehabilitation, Ophthalmology, Genetics, Maternal and Child Health, University of Genoa, Genoa, Italy; 2 Medical Genetics Unit, IRCCS Istituto Giannina Gaslini, Genoa, Italy; 3 Department of Neurology, Houston Methodist Research Institute, Houston, TX 77030, USA; 4 Neuroradiology Unit, IRCCS Giannina Gaslini Institute, 16147 Genoa, Italy; 5 Pediatric Neurology and Muscular Diseases Unit, IRCCS Istituto Giannina Gaslini, Genoa, Italy; 6 U.O.S.D. Centro Traslazionale di Miologia e Patologie Neurodegenerative, Genoa, Italy; 7 Molecular Medicine, IRCCS Fondazione Stella Maris, Pisa, Italy; 8 Department of Neuroscience, Reproductive and Odontostomatological Science, University of Naples “Federico II”, Naples, Italy; 9 Department of Neurology, Weill Cornell Medical College, New York, NY 10065, USA

**Keywords:** *ASAH2*, sphingolipids, ceramide metabolism, ceramidase, neurodevelopment, ophthalmoplegia, cerebellar atrophy, neuropathy, glucosylceramide, Drosophila model

## Abstract

Sphingolipids are integral components of cell membranes and modulate cell survival, proliferation, and apoptosis. *ASAH2* is a brain- and gut-enriched gene encoding the neutral N-acylsphingosine amidohydrolase 2, a poorly characterized member of the human ceramidase family. This enzyme plays a pivotal role in maintaining the sphingolipid homeostasis, which is crucial for neurogenesis and synaptic function in the central and peripheral nervous systems. In fact, a dysregulated sphingolipid metabolism is associated with progressive neurological conditions, including Alzheimer disease and Parkinson disease. Here, we report the identification of biallelic *ASAH2* variants in an individual with a neurodevelopmental condition featuring cognitive impairment, neuropathy, ophthalmoplegia, and progressive cerebellar and extraocular muscles atrophy. Through exome sequencing, we identified very rare missense *ASAH2* variants, predicted to be deleterious by *in silico* analyses. Muscle biopsy histopathologic evaluation revealed features suggestive of neuropathic damage. Lipidomic profiling revealed a hyper-accumulation of glucosylceramide in the subject’s cells. Then, the functional investigation of the *ASAH2* variants in *Drosophila* showed the production of an unstable protein and consistent loss-of-function neuromotor phenotypes. Our findings support *ASAH2* as a candidate gene for a previously uncharacterized neurodevelopmental disorder with neuropathic features and progressive cerebellar atrophy, underscoring the important role of this ceramidase in human nervous systems.

## Graphical abstract

**Figure:**
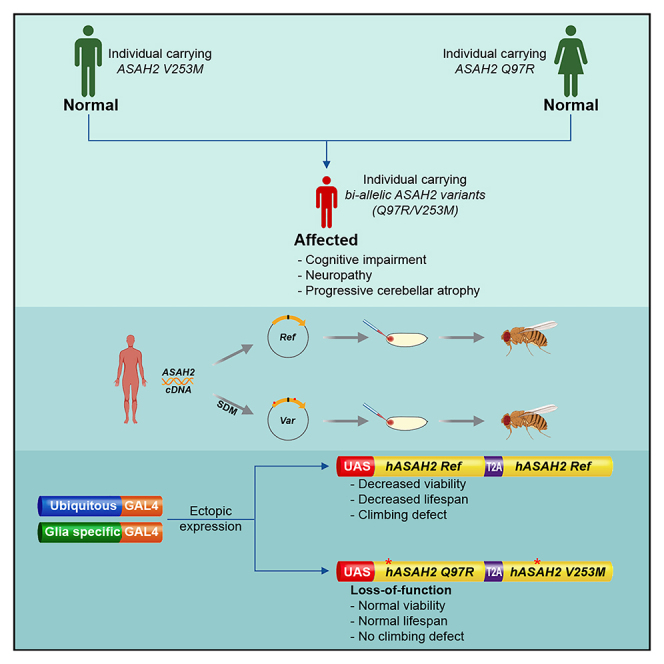

## Teaser

We report rare variants in the gene *ASAH2*, encoding a ceramidase involved in lipid metabolism, in a subject with neurodevelopmental disorder, neuropathy, and cerebellar atrophy. Functional studies revealed disrupted sphingolipid metabolism in human cells and neuromotor disorders in fruit flies. Our work links ASAH2 dysfunction to human nervous system disease.

## Introduction

*ASAH2* (MIM: 611202) encodes the neutral N-acylsphingosine amidohydrolase 2 (ASAH2) enzyme, a poorly characterized member of the ceramidase family, with a pivotal role in sphingolipid metabolism.^1^ The primary function of ASAH2 is to catalyze the hydrolysis of ceramide, a bioactive sphingolipid, into sphingosine and free fatty acids (FFAs).^1^^,^^2^ As such, ASAH2 regulates the ceramide-sphingosine balance.^1^^,^^2^ Additionally, ASAH2 directly contributes to lipid homeostasis and membrane composition, regulating cellular integrity, signaling pathways, and sphingolipid profiles.^2^^,^^3^

Human *ASAH2* is ubiquitously expressed, with higher levels in the gastrointestinal system, pituitary gland, and nervous system (https://gtexportal.org/home/gene/ASAH2). In the brain, *ASAH2* is significantly expressed in areas associated with memory, learning, and emotion regulation, as well as in the cerebellum.^4^ Emerging evidence suggests that this enzyme may play a role in the maintenance of brain function, while sphingolipid dysmetabolism resulting from its deficiency may contribute to the pathogenesis of progressive neurological disorders, such as Alzheimer disease, Parkinson disease, and multiple sclerosis.^4^ Changes in *ASAH2* expression levels have been reported in animal models of these disorders.^4^^,^^5^ However, the association between *ASAH2* dysfunction and human disease remains elusive.

Here, we identified biallelic *ASAH2* variants associated with a neurodevelopmental condition with progressive cerebellar and extraocular muscle atrophy and peripheral neuropathy. Lipidomic profiling in human cells showed glucosylceramide accumulation. Functional assessment in *Drosophila* demonstrated decreased protein expression and abnormal neurobehavioral phenotypes, providing supportive evidence that *ASAH2* variants may contribute to a previously undescribed neurological disorder.

## Material and methods

### Ethics, subject enrollment, clinical assessment, and genetic investigation

The subject was enrolled at the IRCCS Istituto Giannina Gaslini (Genova, Italy) after written informed consent was obtained from the parents. The study was approved by the research ethics committee of Gaslini Children’s Hospital (code 163/2018) and conducted in accordance with the Declaration of Helsinki.

### Genetic and *in silico* analyses

Genomic DNA was extracted using standard procedures.^6^^,^^7^^,^^8^ Array comparative genomic hybridization (aCGH) and trio-based exome sequencing (ES) were performed as previously described.^9^^,^^10^ Raw sequence quality was assessed using FastQC, and reads were aligned to the GRCh38 reference genome with the Burroughs-Wheeler Aligner. Variant calling and realignment were performed using the GATK HaplotypeCaller algorithm.^11^^,^^12^ Annotation was carried out using ANNOVAR,^13^ and variants were filtered for a minor allele frequency (≤0.001) based on the Genome Aggregation Database (gnomAD). The potential functional impact of candidate variants was evaluated using combined annotation-dependent depletion (CADD), MutationTaster, PolyPhen-2, MutationAssessor, and SpliceAI. Confirmatory Sanger sequencing and segregation analyses were performed according to standard protocols.^8^ All variants were annotated based on the ASAH2 RefSeq transcript NM_019893.4 (NP_063946.2). Orthologous information was obtained from the MARRVEL database. Regional intolerance to variation was assessed using Metadome (https://stuart.radboudumc.nl/metadome/), and the effect of ASAH2 variants on protein stability was predicted using DynaMut2.^14^

### Lipidomics

Lipidomics was performed on peripheral blood mononuclear cells using high-resolution liquid chromatography-tandem mass spectrometry to quantify ceramide species. Lipids were extracted with ethyl acetate/isopropanol/water (60:30:10, v/v) containing internal standards, evaporated, and reconstituted in methanol. Chromatographic separation was achieved on a BDS Hypersil C8 column, and peaks were processed using Xcalibur software. Quantification was performed using calibration curves generated with synthetic standards, normalized to internal standards and phosphate content.

### Transgenesis and *Drosophila* assays

Flies were reared on standard cornmeal-molasses medium at 24°C ± 1°C. Transgenic lines expressing upstream activating sequence-ASAH2 reference or variant (p.Q97R + p.V253M) constructs were generated using a *Drosophila*-optimized T2A bicistronic system to ensure coexpression.^15^ Integration was achieved via attB/attP recombination, and transformants were balanced on the third chromosome. Expression was driven by actin-GAL4 (ubiquitous) or repo-GAL4 (glial-specific) drivers.^16^ Viability, lifespan, climbing, and bang-sensitivity assays were performed as described previously, with at least 30 flies per genotype. Total RNA was isolated from larvae using the Monarch Total RNA Kit (NEB), reverse-transcribed to cDNA, and analyzed by quantitative reverse-transcription PCR (RT-qPCR) using RP49 as the reference gene. For western blotting, proteins were separated by SDS-PAGE, transferred to polyvinylidene fluoride membranes, and probed with anti-ASAH2 antibody (Abcam, 1:1,000) and β-tubulin controls. All data were analyzed using GraphPad Prism 10. Results are shown as mean ± SEM. Statistical significance was assessed by two-tailed Student’s *t* test, with *p* < 0.05 considered significant.

## Results

### Clinical data

**Figure 1:**
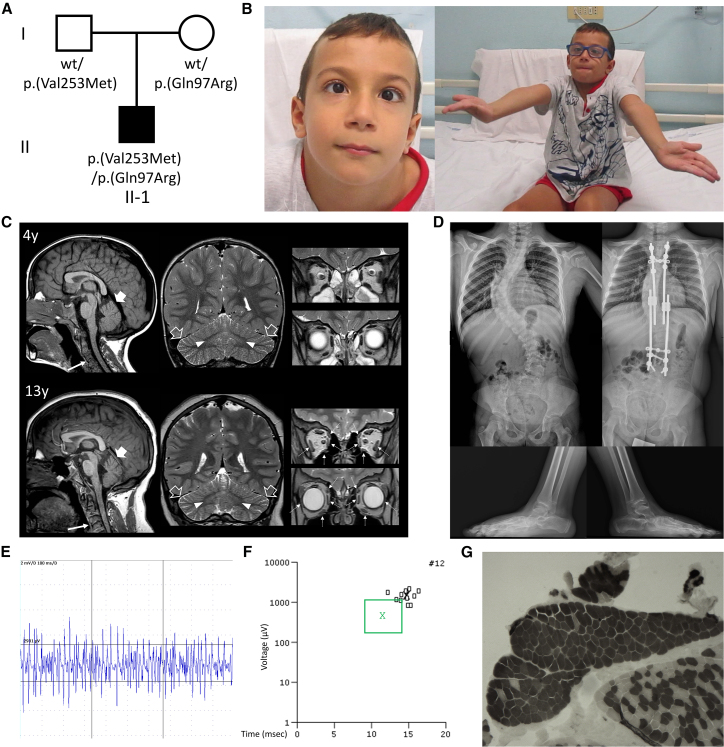
Genetic and clinical findings in the reported subject (A) Pedigree of the family. Pedigree of the reported family showing the segregation of the variants with the neurodevelopmental phenotype in the proband and the carrier status of the unaffected parents. (B) Clinical photographs of the subject. At the age of 7 years, the subject showed mild cognitive impairment, strabismus, decreased strength with tremors in the upper limbs, dysmetria, poor balance, and wide-based gait. (C) Brain MRI scans with sagittal T1-weighted (first column) and coronal T2-weighted (second and third columns) images performed at the ages of 4 and 13 years. There is a global and slowly progressive decrease in the volume with enlargement of cerebellar folia of the cerebellar hemispheres (empty arrows) and superior vermis (thick arrows). In addition, there is progressive atrophy with T2-hyperintensity of the mesial, lateral, and inferior recti muscles and oblique muscles (dashed arrows). Note the C2-C3 fusion in the cervical spine (thin arrows). (D) Skeletal X-rays performed at the age of 7 years. There is severe scoliosis, with Cobb angle >60°, which was corrected through surgical arthrodesis. The subject also showed bilateral pes planus. (E) Needle electromyography showed in tibialis anterior muscle reduced motor unit recruitment at maximum effort. (F) Diagram plot of tibialis anterior muscle motor unit potentials (MUPS). Patients’ MUPS (small black squares) have increased amplitude and duration compared with average MUPS from normal controls (green box). (G) Muscle biopsy at the age of 7 years. Adenosine triphosphate staining shows type grouping, suggesting initial muscular damage secondary to peripheral neuropathy.

The proband is a 14-year-old male born at term to nonconsanguineous healthy parents of Italian ancestry (Figure 1A). Family history was unremarkable. Pregnancy and neonatal course were uneventful. At 7 months, he showed a lack of head control and laxity. Physical examinations at 3 years revealed severe and generalized hypotonia, ataxia, decreased muscle mass, and areflexia. At 7 years, neurological assessments showed mild cognitive impairment, strabismus, limited unsupported ambulation, and cerebellar manifestations, including dysmetria, poor balance, and wide-based gait (Figure 1B; Videos S1, S2, and S3). Ophthalmologic evaluation revealed bilateral ptosis, ophthalmoplegia, and hypermetropia. Brain MRI at the age of 1.5 years showed T2 hyperintensity of the dentate nuclei extending medially to the cerebellar vermis white matter (Figure 1C). Segmentation defects with partial posterior body fusion were observed in C3-C4 cervical vertebrae. Follow-up brain MRI at 4, 7, and 13 years revealed progressive enlargement of cerebellar folia, suggesting mild cerebellar atrophy, and unchanged dentate nuclei alterations. Progressive atrophy of extraocular muscles (mesial, inferior and lateral rectus muscles, and obliques) was noted bilaterally (Figure 1C). Skeletal X-ray showed severe scoliosis, pes pronatus valgus, enlarged metaphyses, and dysmorphic distal femoral epiphyseal nuclei (Figure 1D). Nerve conduction velocities and somatosensory evoked potentials were within normal ranges. However, needle electromyography (EMG) showed reduced motor unit recruitment at maximum effort (Figure 1E), with chronic neurogenic rearrangement of motor units (Figure 1F). Muscle biopsy revealed fiber type grouping confirming neurogenic muscle damage in the absence of respiratory chain defects (Figure 1G). Together with severe hypotonia and areflexia, these EMG findings were suggestive of motor neuronopathy.

Video S1. There is decreased strength in the upper limbs, leading to intentional tremors when the subject (aged 7 years) is asked to extend both arms upright Cerebellar examination reveals diadochokinesia and dysmetria. The subject also shows dyskinetic features in the lower limbs.

Video S2. Extraocular muscle function test shows that the subject (aged 7 years) has an overall limitation in pursuit movements, especially marked for movements to the left

Video S3. The subject (aged 7 years) is able to walk with support He shows wide-based gait and dysmetria. He displays very poor balance and shows dyskinetic features, more marked in the lower limbs.

### Genetic findings

Through ES, we detected bi-allelic *ASAH2* variants in the compound heterozygous state, inherited in *trans*: the paternal c.757G>A, p.(Val253Met) and the maternal c.290A>G, p.(Gln97Arg) (Figure 1A). These variants are absent in gnomAD (version 4.1.0) and in our in-house database of 7,500 control exomes. The p.(Val253Met) and p.(Gln97Arg) affect highly conserved residues, with Genomic Evolutionary Rate Profiling (GERP) scores of 4.6799 and 5.4299, respectively. They are both predicted to be deleterious *in silico* (Table S1). The variants have been deposited in the LOVD database with the accession numbers 0000931620 and 0000931621. No additional candidate variants or copy-number variations in disease-causing genes were detected, including variants in mitochondrial DNA.

**Figure 2:**
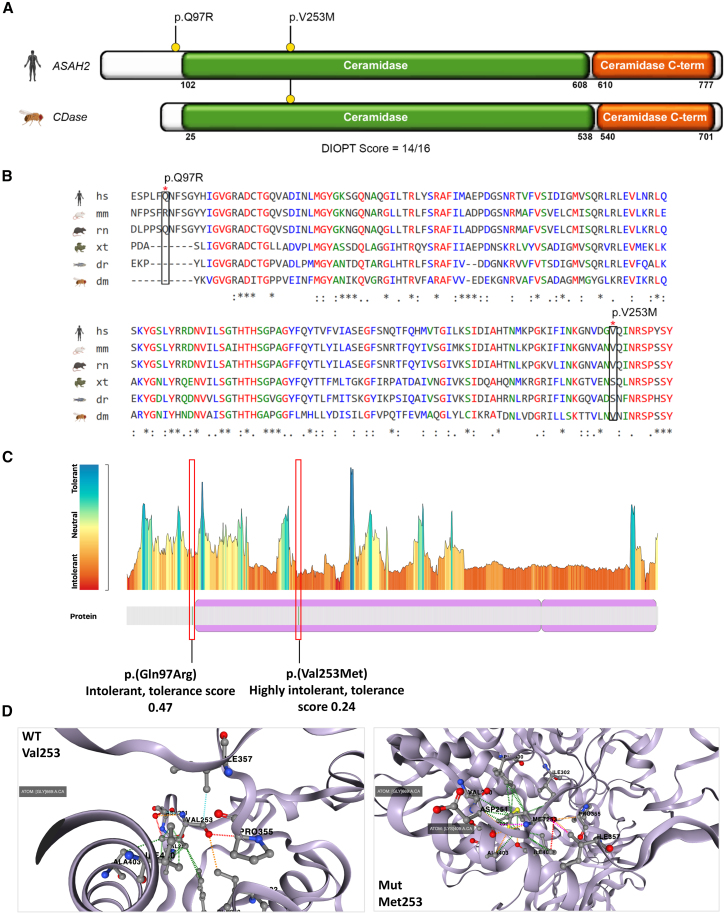
The functional ceramidase domains of ASAH2 are conserved in flies and exhibit a loss of function constraint (A) Schematic drawing of the ASAH2 enzyme (NP_063946.2, neutral ceramidase isoform a) showing the localization of the p.(Gln97Arg) and p.(Val253Met) variants (indicated with yellow circle with black lines) within the long N-terminal neutral/alkaline non-lysosomal ceramidase domain, which is crucial for the catalytic activity of ASAH2. This N-terminal domain carries two metal-binding sites: the first (for Zn^2+^) resides within the domain, and the second (for Mg^2+^/Ca^2+^) lies at the interface between the two domains. The protein domain organization of human ASAH2 is flanked by its fly ortholog (ceramidase; DIOPT score = 14, DRSC Integrative Ortholog Prediction Tool). (B) Protein sequence alignment of human ASAH2 with other species, including *Drosophila*. The variants are highlighted in black borders. (C) Analysis of intolerance to variation according to Metadome. Gln97 is predicted to show a moderate-to-severe intolerance to variation (tolerance score = 0.47), whereas Val253 is predicted to be highly intolerant to variation (tolerance score = 0.24). (D) Protein modeling using DynaMut2 shows that the presence of Met253 affects the tridimensional structure of the protein in a highly functionally relevant region and has a deleterious effect on protein stability, with a ΔΔGStability of −0.64 kcal/mol compared to the wild type (WT).

The fly ortholog neutral ceramidase gene (*CDase* encodes a protein with an overall 72% identity and 84% similarity to human ceramidase (Figures 2A and 2B). These proteins share a highly conserved CDase domain, with 88% and 92% identity for the ceramide C-terminal domain, respectively (Figures 2A and 2B). While Gln97 is only conserved in rats, Val253 is conserved in mouse, *Drosophila*, *Xenopus*, and worm (Figure 2C). Both residues are intolerant to variation and lie within or in proximity to the long N-terminal ceramidase domain (Figure 2C). While no prediction on stability was available for p.(Gln97Arg), Gln97 lies next to N-glycosylation (Asn98) and phosphorylation (Ser100) sites, suggesting a potential impact on post-translational processing (Figure S1). According to DynaMut2,^14^ the p.(Val253Met) may destabilize the protein with a (ΔΔGStability) of −0.64 kcal/mol (Figure 2D). As such, *ASAH2* variants are likely to perturb enzymatic function or interfere with correct folding.

### ASAH2 variants affect ceramide catabolism in human cells

**Figure 3:**
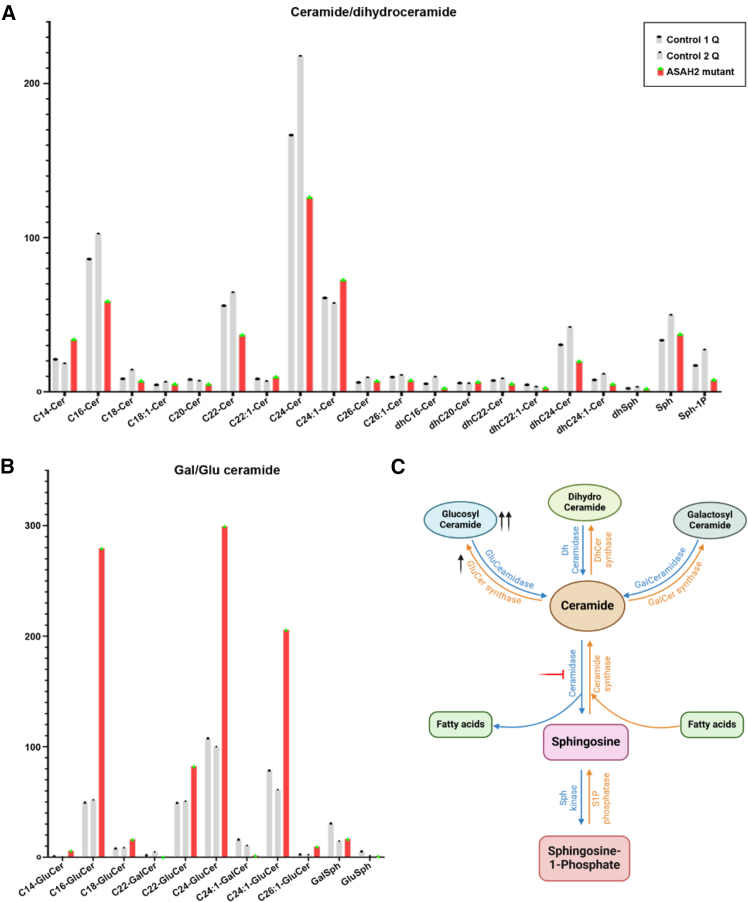
Comparative analysis of ceramide levels in the subject’s cells and controls (A) Bar graphs showing non-significant difference in ceramide levels in two controls and one subject sample. (B) Concentration of glucosylceramide was highly increased in the subject sample (red) irrespective of their carbon chain length. (C) Possible mechanism in ceramide metabolic pathway showing the accumulation of glucosylceramide in the affected individual. DhCer, dihydroceramide; GalCer, galactosylceramide; GluCer, glucosylceramide; S1P, sphingosine-1-phosphate; Sph, sphingosine.

The analysis of the peripheral blood mononuclear cells of the individual did not show significant alterations in ceramide and dihydroceramide levels (Figure 3A). However, we observed a significant accumulation of glucosylceramides (Figure 3B), suggesting that *ASAH2* variants may impair ASAH2 function, although enzymatic activity was not directly measured. ASAH2 is involved in the hydrolysis of ceramides into sphingosine and FFAs. Thus, its loss is expected to disrupt physiological ceramide catabolism, leading to the conversion of excess ceramides into glucosylceramides (Figure 3C). While the presence of normal ceramide levels suggests that compensatory mechanisms may contribute to maintain overall ceramide homeostasis, these results support the pathogenicity of *ASAH2* variants.

### ASAH2 variants negatively affect protein stability

**Figure 4:**
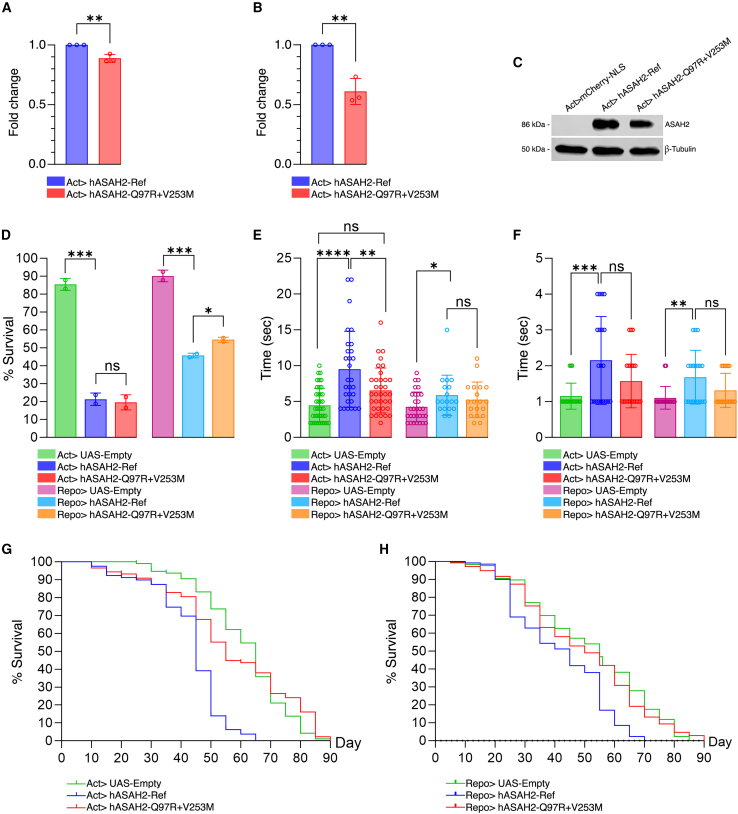
Ectopic expression of *ASAH2* variants in *Drosophila* results in loss of function phenotypes (A) Quantitative real-time PCR result showing modest downregulation in the transcript level of the *ASAH2* variant allele. (B and C) (B) Bar graph and (C) western blot image showing significantly decreased level of ASAH2 protein during variant expression. (D) Viability is not affected by ubiquitous overexpression of *ASAH2* variant as compared with the reference allele. However, significant differences emerge when the variant allele is overexpressed in glial cells, with a decreased toxicity compared with the reference allele. (E) Significant delay in climbing time at day 25 for ubiquitous (not glial-specific) overexpression of *ASAH2* reference compared with the variant allele. (F) No significant difference observed in bang sensitivity between *ASAH2* reference and variant alleles at day 25 when overexpressed ubiquitously or in glial cells. (G and H) Lifespan is highly decreased when the *ASAH2* reference allele is overexpressed ubiquitously or in glia, but it is not affected by variant overexpression. ∗*p* < 0.05; ∗∗*p* < 0.01; ns, not significant.

To assess the impact of *ASAH2* variants on expression, total RNA and protein were extracted from wild-type and mutant flies. Variant-expressing flies showed a 12% reduction in transcript levels (Figure 4A) and a 40% decrease in protein abundance (*p* < 0.01; Figures 4B and 4C). These results indicate that *ASAH2* variants are associated with reduced transcript and protein abundance in this overexpression model, consistent with a potential loss of function (LoF) effect.

### Biallelic *ASAH2* variant is LoF constrained

We next assessed the impact of *ASAH2* variants on viability. Overexpression of the reference allele, either ubiquitously or in glial cells led to significantly reduced viability, reflecting intrinsic toxicity of *ASAH2* overexpression. Similarly, ubiquitous overexpression of the variant allele also caused reduced viability. However, glial-specific expression of the variant resulted in a milder reduction in viability compared to the reference (Figure 4D). This observation is consistent with a partial LoF effect, although this model does not constitute an LoF assay.

To investigate neuromotor function, we performed the negative geotaxis (climbing) assay. Flies expressing the reference allele exhibited significantly impaired climbing ability by day 25, while no significant difference was found in 5-day-old flies regardless of expression pattern (Figure 4E). In contrast, flies expressing the variant allele showed no climbing defects, regardless of expression pattern. This suggests reduced functional activity relative to the wild-type allele, although the precise mechanism remains to be established. These findings reinforce the LoF nature of *ASAH2* variants, emphasizing their inability to support neuromotor performance.

We further investigated neural excitability and seizure susceptibility, using the bang-sensitivity assay. On days 5 and 25, there are no significant differences in seizure susceptibility observed between flies expressing the *ASAH2* reference and those expressing the variant allele, whether expressed ubiquitously or in glial cells (Figure 4F). As such, while overexpression of wild-type *ASAH2* impairs motor function, it does not alter seizure susceptibility, suggesting a specific role of ASAH2 in neuromotor regulation rather than excitability.

Finally, we assessed the impact of *ASAH2* variants on lifespan. Lifespan analysis revealed a marked reduction in survival for flies overexpressing the reference allele, either ubiquitously or specifically in glial cells (Figures 4G and 4H). This likely reflects cumulative deleterious effects of altered sphingolipid metabolism driven by increased ceramidase activity, potentially depleting ceramide pools or causing excessive production of sphingosine. In contrast, overexpression of the variant had no significant impact on lifespan. This suggests that the mutant allele does not reproduce the effects observed with the wild-type allele, consistent with reduced functional activity.

## Discussion

Ceramides play critical roles in neurodevelopment.^17^^,^^18^ Modulating ceramide levels, and thereby influencing S1P and sphingosine levels, ASAH2 may regulate neural stem cell fate, vesicle exocytosis, and synaptic plasticity.^17^^,^^19^^,^^20^ In the peripheral nervous system, *ASAH2* can support Schwann cell development and promote axonal regeneration.^21^ Elevated ceramide levels may correlate with epileptogenesis, and dysregulated ceramidase activity can cause severe neurological syndromes, such as the *ASAH1*-related Farber disease (MIM: 228000).^22^^,^^23^ Additionally, sphingolipid homeostasis defects are involved in neurodegenerative disorders and neuropathies.^22^^,^^23^^,^^24^ Thus, the progressive neurological manifestations associated with *ASAH2* variants may relate to alterations in the ceramide/sphingosine balance and the accumulation of neurotoxic sphingolipid metabolites.^20^ Skeletal manifestations can be secondary to neurological defects or be caused by sphingolipid dysmetabolism. For instance, peripheral motor neuropathy can cause scoliosis due to chronic hypotonia, asymmetric weakness, and impaired postural control,^25^ whereas anomalous sphingolipids levels during bone remodeling and cartilage repair can cause other skeletal defects.^26^^,^^27^

In human cells, *ASAH2* variant expression was associated with increased levels of glucosylceramide, a cerebroside that accumulates and drives neuron loss in neuronopathic Gaucher disease (nGD).^28^ Elevated ceramide levels due to ASAH2 defects can stimulate the activity of glucosylceramide synthase, the enzyme catalyzing the conversion of ceramides into glucosylceramide, which may help explain the increased glucosylceramide levels. Indeed, the cellular machinery may actively channel the excess ceramides toward glycosylation pathways, causing increased production of glucosylceramides. Although this shift may be adaptive to avoid the cytotoxic effects of ceramide accumulation,^29^ it can eventually lead to toxicity due to the accumulation of glucosylceramide, as seen in nGD.^28^ Potential compensatory mechanisms may participate in this complex balance, and further studies will be critical to investigate the underlying regulatory pathways involved in human disease.

*ASAH2* is likely to be constrained for recessive LoF variants (Table S2). In fact, despite the low intolerance to LoF (probability of LoF intolerance score = 0.0) and missense changes (*Z* score = 1.083) (gnomAD, version 4.1.0), the gene shows an LoF variant observed/expected ratio of 0.838 and an observed/expected ratio for missense changes of 0.893, with a probability of falling into distribution of unconstrained (pLoF tolerant) or recessive (pLOF recessive) genes of 11.87% and 87.7%, respectively. This aligns with the results of our studies in *Drosophila*. Flies overexpressing the wild-type allele showed a significant reduction in viability and lifespan, reflecting the toxicity of exogenous protein overexpression.^30^ Additionally, they had significantly impaired motor functions, supporting *ASAH2* involvement in neuromotor regulation. The lack of detrimental effects in mutant flies is consistent with reduced functional activity of the variant allele in this overexpression model, as suggested by decreased transcript/protein abundance and behavioral readouts, although the precise mechanism remains untested and should be interpreted within the known limitations of *Drosophila* overexpression assays.^30^ Interestingly, no significant differences were observed on neural excitability, suggesting that human variants may affect specific neuronal functions (i.e., neuromotor control) regardless of increased epileptogenesis. Comprehensively, our data support a model where two variants act synergistically, behaving as compound heterozygous hypomorphic alleles; each is individually tolerated, but together they cause a deficit exceeding the pathogenic threshold.

Our study has limitations. First, we could not identify other subjects harboring *ASAH2* variants, likely due to the rarity of the disorder or, potentially, the lethality of highly damaging *ASAH2* variants, suggesting that ours act as hypomorphic alleles. Second, in this study our primary aim was to report a previously undescribed association between *ASAH2* and a human disorder. While we provided the functional evidence linking *ASAH2* defects to neuronal anomalies, further validation of *ASAH2* variants in a *CDase*-null/RNAi background would provide a more stringent functional rescue assay. Notably, only a viable CDase CRIMIC (CRISPR-mediated integration cassette) line currently remains available through FlyBase/Bloomington Drosophila Stock Center, and its characterization will be necessary to establish a tractable LoF background for rigorous rescue studies. For this reason, and given the exploratory nature of our study, we focused on overexpression-based assays, while noting that defining the CDase LoF phenotype will be an important future step for variant validation. Third, future experiments evaluating each variant individually using the same matched T2A architecture will be essential to delineate their distinct pathophysiologic contributions. Fourth, while we showed that human variants destabilize functionally critical residues, we did not test ASAH2 enzymatic activity directly. In addition, direct enzymatic assays, such as 4-nitro-2,1,3-benzoxadiazole-ceramide, will be important to quantify their impact on enzymatic function. Fifth, although protein expression in *Drosophila* provides supportive *in vivo* evidence, the mechanisms underlying human disease remain to be fully elucidated, requiring further validation in subjects’ cells.

Here, we report the identification of biallelic LoF variants in *ASAH2* associated with a previously uncharacterized progressive neurological disorder featuring central and peripheral manifestations. We showed that human variants are associated with altered sphingolipid profiles in subjects’ cells and with neuromotor defects in a fly overexpression model, providing supportive evidence for ASAH2 involvement in brain physiology and disease.

## Data and code availability

All data described in this study are provided within the article and supplemental information. The datasets generated during this study are available from the corresponding authors upon reasonable request. No custom code was generated for this study.

## Acknowledgments

We would like to thank the reported individual and his family for their consent and valuable support for our work. H-l.C. is supported by the 10.13039/100002558Warren Alpert Foundation. We acknowledge support from the Mitchell Foundation and start-up funds from the Houston Methodist Academic Institute. M.S. is funded by the 10.13039/501100003196Italian Ministry of Health (grant RF-2016-02361949 to F.Z.) and by 10.13039/100007388Compagnia di San Paolo (grant ROL: 32628 to F.Z.). This research was also supported by PNRR-MUR-M4C2 PE0000006 Research Program “MNESYS,” a multiscale integrated approach to the study of the nervous system in health and disease. IRCCS Istituto Giannina Gaslini is a member of European Reference Network-EpiCARE.

## Declaration of interests

The authors declare no competing interests.

## Web resources

CADD, http://cadd.gs.washington.edu/
ClinVar, https://www.ncbi.nlm.nih.gov/clinvar
DECIPHER, https://decipher.sanger.ac.uk/
DynaMut2, https://biosig.lab.uq.edu.au/dynamut2
Ensembl Variant Effect Predictor (VEP), https://www.ensembl.org/info/docs/tools/vep/index.html
Gene Cards, https://www.genecards.org/
Gene Matcher, http://www.genematcher.org/
GERP, http://mendel.stanford.edu/SidowLab/downloads/gerp/
gnomAD, http://gnomad.broadinstitute.org/
MARRVEL web portal, https://marrvel.org/
Metadome, https://stuart.radboudumc.nl/metadome/dashboard
Mutation Taster, http://www.mutationtaster.org/
NeXtProt, https://www.nextprot.org/
Online Mendelian Inheritance in Man, https://www.ncbi.nlm.nih.gov/Omim
Phosphosite, https://www.phosphosite.org/
Protein Atlas, https://www.proteinatlas.org/ENSG00000188611-ASAH2/brain
PubMed, https://www.ncbi.nlm.nih.gov/pubmed
RefSeq, https://www.ncbi.nlm.nih.gov/refseq
SIFT, https://sift.bii.a-star.edu.sg/
UCSC Human Genome Database, https://www.genome.ucsc.edu/
UniProt, https://www.uniprot.org/
Varsome, https://varsome.com/

## Supplemental information

Document S1. Figure S1, Table S2, and supplemental methods

Table S1. Extended *in silico* analysis of the identified ASAH2 variants

Document S2. Article plus supplemental information
