# Supplementary material for: ASAH2 deficiency affects sphingolipid homeostasis and neuromotor control, causing a progressive neurological disorder
Source: HGG Adv. 2026 Mar 10;7(2):100587. doi: 10.1016/j.xhgg.2026.100587 (PMC13022665; doi:10.1016/j.xhgg.2026.100587)
Supplement: Document S1. Figure S1, Table S2, and supplemental methods [file mmc1.pdf]

**HGGA, Volume 7**

## Supplemental information

# ASAH2 deficiency affects sphingolipid homeostasis and neuromotor control, causing a progressive neurological disorder

**Marcello Scala, Ranjan K. Sahu, Mariasavina Severino, Monica Traverso, Michele Iacomino, Marina Pedemonte, Filippo Santorelli, Stefano Tozza, Federico Zara, Chiara Fiorillo, and Hyung-lok Chung**

# Supplemental information

## Table of Contents

1. Supplemental Figures and Legends 3

2. Supplemental Tables 4

3. Supplemental Methods 5

4. Supplemental References 10

## 1. Supplemental Figures and Legends

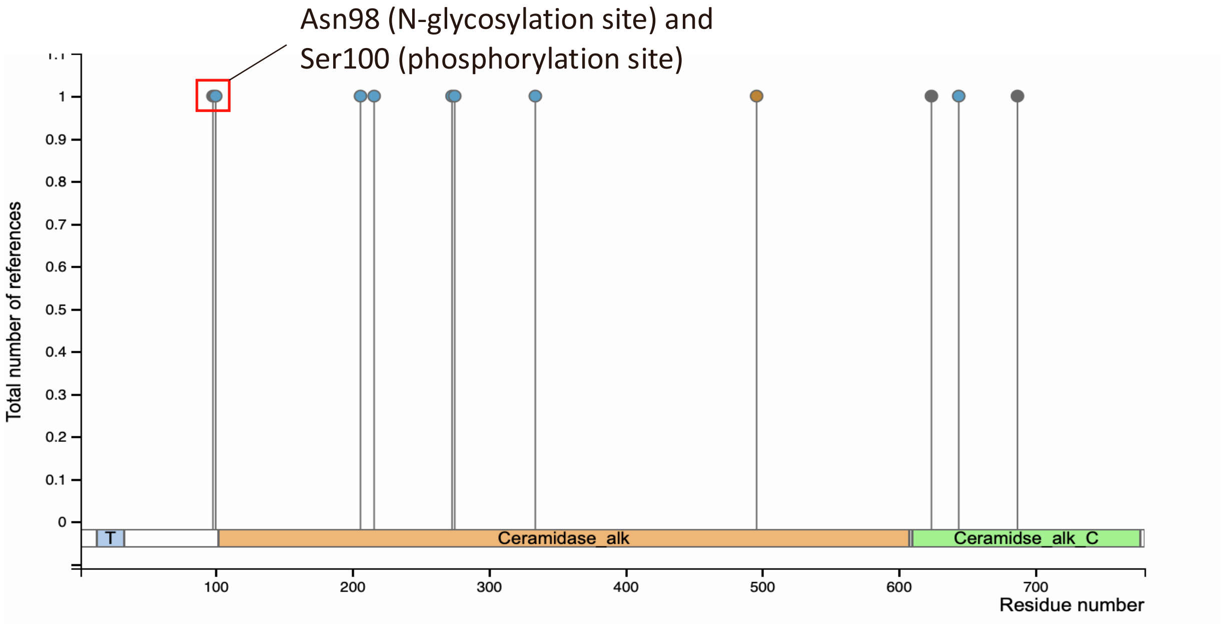


**Figure S1. Prediction on the impact of the p.(Gln97Arg) variant.** Gln97 lies in close proximity to two important sites used for the N-glycosylation (Asn98) and phosphorylation (Ser100) of the ASAH2 protein. The substitution of this residue by Arginine could affect this post-translational modification, with deleterious effects on protein function. Adapted from Phosphosite (https://www.phosphosite.org/).

## Supplemental movie titles and legends

**Movie S1.** There is decreased strength in the upper limbs, leading to intentional tremors when the patient (aged 7 years) is asked to extend both arms upright. Cerebellar examination reveals diadochokinesia and dysmetria. The patient also shows dyskinetic features in the lower limbs.

**Movie S2.** extraocular muscle function test shows that the patient (aged 7 years) has an overall limitation in pursuit movements, especially marked for movements to the left.

**Movie S3.** The patient (aged 7 years) is able to walk with support. He shows wide-based gait and dysmetria. He displays a very poor balance and shows dyskinetic features, more marked in the lower limbs.

## 2. Supplemental Tables

**Table S1. Extended *in silico* analysis of the identified *ASAH2* variants.**

**Table S2. Constraint metrics for *ASAH2*.**

| **Probability of LoF intolerance (pLoF)** | | | | **Missense constraints** | | | | **Domino** | |
|---|---|---|---|---|---|---|---|---|---|
| Expected | Observed | pLi score | O/E score | Expected | Observed | O/E score | z-score | Inheritance prediction | Probability of being AD |
| 71.5 | 47 | 0 | 0.66 | 766.1 | 684 | 0.893 | 1.08 | Very likely recessive | 0.108 |

Constraint metrics according to gnomAD (v4.1.0)[1] and Domino[2].

## 3. Supplemental Methods

### *3.1. Participants enrolment*

The patient investigated in this study was enrolled at the IRCCS Istituto Giannina Gaslini (Genova, Italy), after informed consent was obtained from his parents. For clinical assessment, the patient was assessed by expert pediatric neurologists and medical geneticists. Brain MRI scans were reviewed by a pediatric neuroradiologists with 15 years of experience (MS).

### *3.2. Ethics, subject enrolment, clinical assessment, and genetic investigation*

This study adheres to the Declaration of Helsinki and was approved by the Research Ethics Committees of Gaslini Children's Hospital (code 163/2018). Written informed consent was obtained by the parents. Array comparative genomic hybridization (aCGH) and trio-exome sequencing (ES) were performed as previously described[3,4]. Variants were filtered out according to allele frequency in gnomAD (< 0.001), conservation, and predicted impact on protein function (Supplemental Information)[3,4]. *ASAH2* variants are reported according to the RefSeq transcript NM_019893.4 (corresponding to the isoform of the neutral ceramidase, NP_063946.2). Information about *ASAH2* orthologs was collected from MARRVEL web portal (https://marrvel.org/). Intolerance to variation was analyzed using Metadome (https://stuart.radboudumc.nl/metadome/). Protein modeling was performed to investigate the impact of *ASAH2* variants on protein stability using DynaMut2[5].

### *3.3. Exome sequencing analysis*

After standard DNA extraction, trio-exome sequencing (ES) was performed in all subjects as previously described[6-10]. QC statistics with FastQC (http://www.bioinformatics.bbsrc.ac.uk/projects/fastqc) were used to assess the quality of the sequence reads. BWA with default parameters was used for reads alignment to the reference human genome (GRCh38 - hg38, UCSC genome assembly). Recalibration of the quality score and for indel realignment and variant calling was performed through the HaplotypeCaller algorithm within the GATK package[11,12]. Variants were then annotated with ANNOVAR[13] and filtered out for minor allele frequency (MAF) ≤ 0.01 in genomic databases (GnomAD, https://gnomad.broadinstitute.org). Afterwards, *in silico* tools were employed to predict the impact of candidate variants on protein structure and function, including: Combined Annotation Dependent Depletion (CADD, https://cadd.gs.washington.edu), Mutation Taster (http://www.mutationtaster.org), Mutation Assessor (http://mutationassessor.org/r3/), Polyphen-2 (http://genetics.bwh.harvard.edu/pph2/), and

Splice AI (https://spliceailookup.broadinstitute.org). Sanger sequencing was performed according to standard procedures[10] to confirm the most plausible candidate variants and for parental segregation analysis.

### *3.4. Sanger sequencing*

Candidate variants were validated by Sanger sequencing using High-Fidelity Platinum Master Mix (Thermo Fisher Scientific) for PCR amplification and the BigDye Terminator v1.1 kit (Thermo Fisher Scientific) for sequencing.

### *3.5. Lipidomics profiling*

We utilized lipidomics to analyze ceramide compositions in peripheral blood mononuclear cells of the individual, aiming to identify differentially affected ceramide moieties in blood samples of patient and control. High-resolution mass spectrometry (LC-MS/MS) was employed for identification and quantification of different classes of ceramides, coupled with chromatographic separation to enhance specificity and resolution. Blood samples were collected and fortified with internal standards (C17 base D-erythro-sphingosine, C17 sphingosine-1-phosphate, 13C16-Cer, and C17-Cer). Lipids were extracted using an ethyl acetate/isopropanol/water (60:30:10 v/v) solvent system, followed by evaporation and reconstitution in 100 µL of methanol. The samples were analyzed using an HP1100/TSQ Quantum LC/MS system equipped with a BDS Hypersil C8 column (150 × 3.2 mm, 3 µm particle size) and a gradient mobile phase of methanolic and aqueous ammonium formate. Peaks corresponding to the target analytes and internal standards were processed using Xcalibur software. Quantitation was performed using calibration curves generated with synthetic standards, normalized to internal standard peak area ratios, and analyzed via linear regression. Sphingolipid levels in patient cells were normalized to the phosphate content of the samples. This method provides reliable results for comparative studies of sphingolipid levels in patient-derived cells.

### *3.6. Fly husbandry and fly stocks*

Flies were reared at $24 \pm 1^{0}$C, on vials having standard cornmeal and molasses medium. All transgenic fly lines used in this study were generated or obtained from Bloomington Drosophila Stock Center (BDSC).

### *3.7. Generation of UAS-ASAH2 transgenic lines*

Here, we utilized the ASAH2 mRNA (RefSeq NM_019893.4 / NP_063946.2) encoding the full-length ASAH2 as a template to generate the reference (*UAS-ASAH2 Ref*) and variant (*UAS-ASAH2-p.Q97R + p.V253M*) alleles. We employed the T2A strategy to generate transgenic flies expressing either the reference or a compound heterozygous missense alleles, which were cloned under the *UAS* promoter in a wild-type background, as previously described[14]. Both GOI fragments were de novo synthesized, ns/co codon usage pattern was kept identical between reference and variant double ORF constructs to avoid confounding by codon optimization and assembled into entry clones by Gibson and then transferred by LR recombination into pUASTattB (5×UAS/mini Hsp70). The *Drosophila* optimized T2A (dme) sequence was already present in the PCR template, and after Gibson assembly the two ASAH2 ORFs were seamlessly joined in frame. The 2A peptide mediates co translational "ribosome skipping" at the conserved Gly Pro junction, yielding two independent polypeptides from a single transcript; the upstream product retains the short 2A scar at its C terminus, and the downstream product initiates with Pro, enabling near stoichiometric co expression while minimizing fusion artifacts. To ensure comparable expression levels and to control any 2A architecture effects, the reference construct was built in the same double ORF T2A configuration as the variant construct. The constructs were subsequently injected into $y^1,w^1$ embryos. Positive transformants were identified by screening progeny for the presence of red eyes and balanced with 3$^{rd}$ chromosome balancer. *Actin-GAL4* was used for ubiquitous expression, while *repo-GAL4* was utilized to target expression specifically to glial cells, where *CDase* is predominantly expressed[15].

*3.8. Viability & life span assay*

Freshly enclosed adult flies were separated into plastic vials containing standard cornmeal at a low density (20 flies per vial) and reared at 25$^0$C. Flies were transferred to new food vials every 3 days, and the number of dead flies were recorded. The survival rate was calculated based on the percentage of flies that survived each day until all flies perished. Survival curves were produced and validated using the Log-rank (Mantel-Cox) test.

*3.9. Climbing assays*

For climbing assay, 25-day-old flies were anesthetized 24 h prior to the assay tested and were reared in food vials at room temperature. For the assay, these flies were transferred (without anesthesia) to a transparent cylinder with an 8 cm mark. The flies were tapped thrice on the base of the cylinder to assess negative geotaxis (upward climbing) and observed for 30s and recorded the time taken by each fly to reach to the 8cm mark. A total of 25 to 30 flies were assayed for each genotype.

*3.10. Bang-sensitivity assay*

In order to conduct the Bang-sensitivity assay, the process for fly rearing and anesthetizing is identical to the procedure for the climbing assay. For the assay, a total of 30 flies in three distinct vials were administrated vibration shock (vortexed) for 10 seconds and the duration till they were upright and mobile was recorded. The recovery time cutoff was 30 seconds and were examined for each genotype.

*3.11. qPCR*

A total of 3 biological replicates were performed to isolate the total RNA from 10 larvae using the Monarch Total RNA Miniprep Kit (NEB) and the purity and concentration of RNA were evaluated using a NanoDrop One/OneC Spectrophotometer (Thermo Fischer Scientific). Subsequently, 0.5 µg of total RNA from each individual extraction was subjected to qPCR in triplicate (using the Luna® Probe One-Step RT-qPCR Kit (NEB)) on the CFX Opus Real-Time PCR System (Bio-Rad) with particular primer sets. The housekeeping genes RP49 was used to normalize the relative expression of the target genes. Primer sequences used for these experiments are RP49 Forward-TTGAGAACGCAGGCGACCGT, RP49 Reverse- CGTCTCCTCCAAGAAGCGCAAG, ASAH2 Forward- TGGCCTAGTTCTCCCTAGCAT and ASAH2 Reverse-GGCGAAGAGGGACTTTCACT.

*3.12. Western blotting*

Adult flies of desired genotypes were frozen in -80$^0$C and were homogenized in 150 µl 4X SDS sample loading buffer (Merck) with 1% 2-β-mercaptoethanol and centrifuged at 16,000g at 4°C for 20 min and the supernatant was collected in a fresh tube. A 10 µl of supernatant was run on a 4-20% polyacrylamide gel and then transferred to a PVDF Membrane using Transblot-Turbo transfer system (Bio-Rad). The membranes were blocked with EveryBlot Blocking Buffer (Bio-Rad) at RT and then incubated overnight with ASAH2 antibody (Abcam-ab63804 (1:1000)). The membrane was then rinsed thrice with TBST and incubated with HRP-conjugated secondary antibody (1:5000) and again rinsed thrice with TBST. Membranes were then exposed to HRP substrate solution (Clarity Western ECL from Bio-Rad), and images acquired using C-DiGit Blot Scanner (LI-COR). All ASAH2 band intensities were normalized against their corresponding β-tubulin band intensities across three biological replicates and quantitative expression analysis was performed using ImageJ.

*3.13. Data analysis*

GraphPad Prism 10 was used for all kinds of statistical analysis performed in this study. The student's t-test was employed to compare two groups with normally distributed data. A cutoff point of $p < 0.05$ was established for statistical significance. The results are displayed as mean ± standard error of the mean (SEM) for datasets with larger sample sizes, including neurobehavioral analyses.

## 4. Supplemental References


1. Karczewski, K.J., Francioli, L.C., Tiao, G., Cummings, B.B., Alföldi, J., Wang, Q., Collins, R.L., Laricchia, K.M., Ganna, A., Birnbaum, D.P., et al. (2020). The mutational constraint spectrum quantified from variation in 141,456 humans. Nature *581*, 434-443. 10.1038/s41586-020-2308-7.
2. Quinodoz, M., Royer-Bertrand, B., Cisarova, K., Di Gioia, S.A., Superti-Furga, A., and Rivolta, C. (2017). DOMINO: Using Machine Learning to Predict Genes Associated with Dominant Disorders. American journal of human genetics *101*, 623-629. 10.1016/j.ajhg.2017.09.001.
3. Nishikawa, M., Scala, M., Umair, M., Ito, H., Waqas, A., Striano, P., Zara, F., Costain, G., Capra, V., and Nagata, K.I. (2023). Gain-of-function p.F28S variant in RAC3 disrupts neuronal differentiation, migration and axonogenesis during cortical development, leading to neurodevelopmental disorder. Journal of medical genetics *60*, 223-232. 10.1136/jmedgenet-2022-108483.
4. Scala, M., Accogli, A., De Grandis, E., Allegri, A., Bagowski, C.P., Shoukier, M., Maghnie, M., and Capra, V. (2018). A novel pathogenic MYH3 mutation in a child with Sheldon-Hall syndrome and vertebral fusions. American journal of medical genetics. Part A *176*, 663-667. 10.1002/ajmg.a.38593.
5. Rodrigues, C.H.M., Pires, D.E.V., and Ascher, D.B. (2021). DynaMut2: Assessing changes in stability and flexibility upon single and multiple point missense mutations. Protein science : a publication of the Protein Society *30*, 60-69. 10.1002/pro.3942.
6. Aspromonte, M.C., Bellini, M., Gasparini, A., Carraro, M., Bettella, E., Polli, R., Cesca, F., Bigoni, S., Boni, S., Carlet, O., et al. (2019). Characterization of intellectual disability and autism comorbidity through gene panel sequencing. Hum Mutat *40*, 1346-1363. 10.1002/humu.23822.
7. Bowling, K.M., Thompson, M.L., Amaral, M.D., Finnila, C.R., Hiatt, S.M., Engel, K.L., Cochran, J.N., Brothers, K.B., East, K.M., Gray, D.E., et al. (2017). Genomic diagnosis for children with intellectual disability and/or developmental delay. Genome medicine *9*, 43. 10.1186/s13073-017-0433-1.
8. Harripaul, R., Noor, A., Ayub, M., and Vincent, J.B. (2017). The Use of Next-Generation Sequencing for Research and Diagnostics for Intellectual Disability. Cold Spring Harbor perspectives in medicine *7*. 10.1101/cshperspect.a026864.
9. Murdock, D.R., Dai, H., Burrage, L.C., Rosenfeld, J.A., Ketkar, S., Müller, M.F., Yépez, V.A., Gagneur, J., Liu, P., Chen, S., et al. (2021). Transcriptome-directed analysis for Mendelian disease diagnosis overcomes limitations of conventional genomic testing. The Journal of clinical investigation *131*. 10.1172/jci141500.
10. Tarailo-Graovac, M., Shyr, C., Ross, C.J., Horvath, G.A., Salvarinova, R., Ye, X.C., Zhang, L.H., Bhavsar, A.P., Lee, J.J., Drögemöller, B.I., et al. (2016). Exome Sequencing and the Management of Neurometabolic Disorders. The New England journal of medicine *374*, 2246-2255. 10.1056/NEJMoa1515792.
11. DePristo, M.A., Banks, E., Poplin, R., Garimella, K.V., Maguire, J.R., Hartl, C., Philippakis, A.A., del Angel, G., Rivas, M.A., Hanna, M., et al. (2011). A framework for variation discovery and genotyping using next-generation DNA sequencing data. Nature genetics *43*, 491-498. 10.1038/ng.806.
12. McKenna, A., Hanna, M., Banks, E., Sivachenko, A., Cibulskis, K., Kernytsky, A., Garimella, K., Altshuler, D., Gabriel, S., Daly, M., and DePristo, M.A. (2010). The Genome Analysis Toolkit: a MapReduce framework for analyzing next-generation DNA sequencing data. Genome research *20*, 1297-1303. 10.1101/gr.107524.110.
13. Wang, K., Li, M., and Hakonarson, H. (2010). ANNOVAR: functional annotation of genetic variants from high-throughput sequencing data. Nucleic acids research *38*, e164. 10.1093/nar/gkq603.
14. Chung, H.L., Wangler, M.F., Marcogliese, P.C., Jo, J., Ravenscroft, T.A., Zuo, Z., Duraine, L., Sadeghzadeh, S., Li-Kroeger, D., Schmidt, R.E., et al. (2020). Loss- or Gain-of-Function Mutations in ACOX1 Cause Axonal Loss via Different Mechanisms. Neuron *106*, 589-606.e586. 10.1016/j.neuron.2020.02.021.
15. Chung, H.L., Ye, Q., Park, Y.J., Zuo, Z., Mok, J.W., Kanca, O., Tattikota, S.G., Lu, S., Perrimon, N., Lee, H.K., and Bellen, H.J. (2023). Very-long-chain fatty acids induce glial-derived sphingosine-1-phosphate synthesis, secretion, and neuroinflammation. Cell metabolism *35*, 855-874.e855. 10.1016/j.cmet.2023.03.022.
